# Research on Sentiment Analysis and Satisfaction Evaluation of Online Teaching in Universities During Epidemic Prevention

**DOI:** 10.3389/fpsyg.2021.738776

**Published:** 2021-10-18

**Authors:** Jing Zhang

**Affiliations:** School of Mechanical Technology, Wuxi Institute of Technology, Wuxi, China

**Keywords:** sentiment analysis, online teaching, satisfaction evaluation, fuzzy Bayesian theory, evaluation index system of online teaching

## Abstract

Sentiment analysis of online and offline integrated teaching in universities is being paid more and more attention. Many universities have carried out online teaching activities. However, due to the lack of face-to-face teaching, the lack of emotional communication is the key problem affecting the quality of online teaching. We analyze the relations from the perspectives of the change of teaching mode, the reconstruction of teacher-student relationship, and the transmission of emotional attitude of teachers and students in this paper. Then based on the Bayesian network (BN) theory, the satisfaction of online teaching can be evaluated from the aspects of emotion analysis, learning investment, and teaching interaction. Further, some suggestions are put forward to improve the satisfaction of online teaching.

## Introduction

In 2020, the COVID-19 epidemic hit the world. It affected the normal life of people everywhere and changed the teaching mode of traditional higher education seriously. China’s Ministry of Education had encouraged schools at all levels to make use of various online platforms to conduct online teaching. Subsequently, Chinese universities, primary and secondary schools have launched online education before other countries.

Online teaching is the main way for students to study independently during the epidemic period. As the subject of education, students have had to quickly adapt to the new teaching mode. The change from the traditional offline teaching mode requires teachers to combine online and offline flexibly and realize the seamless transition between teaching and learning, which creates high requirements for the comprehensive teaching ability of teachers. In online teaching, because teachers teach across the screen, students can’t directly feel the presence of teachers. This mode leads to a lack of good emotional communication with teachers, which reduces the investment and participation of students. This has a great impact on the online teaching. In addition, the teaching platform and its upgrading will affect the satisfaction evaluation of online teaching. Therefore, online teaching satisfaction is evaluated from the aspects of emotion analysis, learning investment, and teaching interaction. Then based on the Bayesian network (BN) model, some suggestions with strong operability are proposed.

Scholars have achieved a lot of research results on sentiment analysis and the satisfaction of online teaching. A comprehensive evaluation of online teaching satisfaction was evaluated based on the text mining method (Zhang et al., 2019). A complete mental health evaluation system is constructed based on information technology. This system can help us to understand the attitudes, opinions, and emotions of students. And we can observe their mental health state under the change of emotion (Zhou et al., 2020). In order to analyze the public opinions of “Weibo” users on online learning, web crawler software was used to crawl relevant blog data on Weibo. A text analysis method was applied to the obtained public opinion information to explore the emotional tendency of the communication of online public opinions (Liu and Yue, 2019; Guo and Min, 2020). The harmonious relationships between the teachers and the students can help to promote positive classroom interactions. A good teacher-student relationship can improve the effectiveness of classroom teaching (Nie, 2008). In order to find out the affective tendency in student teaching evaluation and provide a scientific basis for improving teaching quality, a deep learning model was proposed, based on a double-channel deep memory network for sentiment analysis in student teaching evaluation (Wang et al., 2021). A new method based on a krill swarm optimization support vector machine (KH-SVM) was proposed to evaluate the performance of college students, which was affected by the penalty coefficient and kernel function parameters (Li and Wang, 2021).

## Sentiment Analysis of Online Teaching

### The Significance of Emotional Resonance in Teaching

The teaching modes of universities were mostly offline teaching before the COVID-19 epidemic. Teaching processes in which teachers create a harmonious teaching environment are advantageous to the students. Teachers and students can properly handle the relationship between emotion and cognition in the teaching process, and teaching modes in face-to-face teaching give full play to the positive role of emotional factors and enhance students’ positive emotional experience by emotional communication. So it can cultivate and develop students’ emotions, then stimulate their thirst for knowledge and spirit of exploration. This teaching method urges them to form independent and sound personalities and characteristics. Teachers can shorten the distance with students in language, psychology, and space in classroom teaching. They deal with, adjust, guide, and control the psychological relationship between teachers and students correctly so that students can have a kind, pleasant, and positive emotional experience. So a teaching mode of face-to-face teaching is conducive to the formation of a good and harmonious education environment full of emotions and promotes the virtuous cycle of teaching and learning.

### Lack of Emotional Input in Offline Teaching

Man is different from all other creatures because people have emotions. As far as the present situation is concerned, the channels and ways that most live broadcasting platforms provide for teachers and students to interact with each other are limited. But that is not an excuse for teachers to ignore the emotional aspects of online teaching. In online learning, teachers and students are usually separated by a computer screen. Students can see the teacher, but the teacher can’t see all the students at once. With no audience in sight, the teacher is like a soloist standing in the wilderness. They easily lose the passion for teaching and feel that time stands still: their mind becomes stagnant, and they may even become incoherent. The teachers’ performance is likely to directly affect the students’ interest in listening to the class and thereby form a vicious circle.

In addition, there are many differences in the teaching platform, network environment, and teaching methods. During online teaching across the screen, students suffer from a lack of emotional communication with the teacher. They cannot raise awareness of their participation and cooperation and can only accept knowledge mechanically. In the network classroom, the students quietly lose interest. If truancy occurs in large numbers, then the teacher’s efficacy will be greatly reduced, so that teachers and students cannot achieve emotional resonance in teaching activities. In such an atmosphere or teaching situation, the emotions of teachers and students cannot be highly consistent in mutual feeling, interaction, and mutual motivation.

In the context of online teaching, the selection of teaching content needs to be informed by science, especially in ensuring that the presentation form of the content is suitable for the online teaching mode. Because of their digital nature, network classrooms are a carrier with infinite memory ability. All the language and behavior of the teacher, as well as the subtle emotional reactions of the audience, can be recorded on the network. For a teacher, a casual mistake may be magnified on the internet, or even fermented into a storm of public opinion, which will bring great pressure or even negative influence to the teacher and the school.

People’s normal and well-adapted lives had been disrupted by COVID-19, and many are facing mental health challenges. Mental health criteria are observed, including cognitive health, emotional health, personality health, social function, and psychological adjustment. During the COVID-19 outbreak, students’ mental health often manifest anxiety, fear, depression, despair, anger, etc., and their emotions are more volatile than usual. Teachers’ mental health problems are reflected in the disruption of life and work rhythms. There are insufficient boundaries between work and home for teachers. In addition, they have less experience with online tools from before the COVID-19 outbreak. They worry about the poor network connection, incorrect operation, and students’ lack of concentration. In online classes, teachers face not only students but also parents and even more people, which increases the pressure on teachers.

In conclusion, emphasis on scientific and emotional elements in online teaching is very important for all teachers and universities. The scientific design of online teaching will enable students to feel the beauty of science and emotion so that online teaching of science can become an art and a kind of enjoyment, and online teaching can become a new teaching method that complements offline teaching.

## Online Teaching Evaluation Research

Compared with traditional offline teaching, online teaching is not just a change of location. In online teaching, the learning environment, learning methods, teacher-student relationships, and teaching management methods have all changed. Online teaching satisfaction is closely related to students’ learning ability, teachers’ comprehensive quality, the network environment and hardware equipment, etc. Therefore, the evaluation of online teaching satisfaction needs to be comprehensively analyzed from multiple perspectives.

### Data Sources

In order to fully understand the actual situation and satisfaction of online teaching during the epidemic period, 1,180 students from some colleges and universities in Jiangsu are surveyed by questionnaire. The survey subjects include higher vocational colleges, vocational colleges, and undergraduate universities. Survey data show that teachers choose a variety of online teaching platforms and teaching methods. At the same time, in the investigation and research of this paper, the satisfaction of some college students in Jiangsu Province with regard to online teaching during the epidemic period is also counted.

### Online Teaching Satisfaction Analysis

(1) Students’ online learning status is poor. According to the survey data, only 47% of students are satisfied with their learning status. The learning environments in students’ homes are not as good as in school, and their psychologies are affected by the epidemic, so their learning status is negatively affected. Their satisfaction with online teaching is affected by these problems.

(2) Students have high satisfaction with teachers’ teaching attitudes. In the survey, 81% of the students have a good satisfaction with teachers’ teaching attitudes. Students know clearly whether the teachers are serious about their education.

(3) Students lack of self-planning. Scientific self-learning planning is conducive to students’ self-learning, but the survey data show that more than half of the students lack self-learning planning. Only 47% of the students are satisfied with their self-learning planning. Without a clear self-learning plan, the learning efficacy will be reduced to some extent, thus affecting the satisfaction of online teaching.

(4) Students’ low mastery of knowledge. According to the statistics, only 33% of the students have excellent satisfaction with their knowledge mastery. Horizontal analysis shows that students’ satisfaction with the online learning environment, learning state, and self-planning are low. These influencing factors all directly affect students’ knowledge mastery, so most students’ knowledge mastery is not high.

Based on survey data of online teaching satisfaction, we built a multi-index system for evaluating online teaching satisfaction. Using the method of fuzzy empowerment, the probability values of the top indices are accurately computed by combining them with fuzzy Bayesian theory. The proposed method can further infer the weak links of online teaching during the epidemic period and provide a useful reference for the development of online teaching in a future emergency. Finally, some reasonable improvement measures of online teaching are put forward.

### Online Teaching Satisfaction Evaluation Based on Improved Fuzzy Bayesian Network

A BN is a graph network model used to describe the uncertain causal relationship between variables. It is a directed acyclic graph composed of nodes, directed line segments that represent the causal relationship between connected nodes, and probability tables of nodes. A BN combines a directed acyclic graph and probability theory organically and has strong bi-directional reasoning ability. Bayesian networks play a great role in risk analysis and multi-state uncertainty reasoning.

Based on fuzzy BNs and fuzzy weighting methods, this paper improves the fuzzy BN. The object of weighting is the basic-level indexes. After the weighting of basic-level indexes, it plays a role in adjusting the influence of basic-level indexes on middle-level indexes when calculating middle-level indexes, to improve the accuracy of top-level indexes. The weighting method is the expert weighting method. The unified weights are obtained after the experts study the relationships of the basic-level indexes. The evaluation index system after weighting is more appropriate to the actual situation, so the calculated results are more accurate.

## Online Teaching Satisfaction Evaluation Based on Improved Fuzzy Bayesian Network

### Build an Evaluation Index System for Online Teaching Satisfaction

The top-level index T of online teaching satisfaction is determined, and then the influencing factors are extended and analyzed from the three levels including the learning quality *A*, the teaching quality *B*, and the platform management *C*, respectively. The evaluation index system of online teaching satisfaction is obtained, as shown in Table 1.

**TABLE 1:** Evaluation index system of online teaching.

**The top-level index**	**The middle-level index**		**The basic-level index**
	Learning quality *A*	Learning conditions *A*_1_	Learning environment *A*_11_
			Learning facilities *A*_12_
		Learning efficiency *A*_2_	Learning state *A*_21_
			Study plan *A*_22_
		Study achievement *A*_3_	Knowledge mastery *A*_31_
			Degree of completion *A*_32_
Online Teaching Satisfaction *T*	Quality of teaching *B*	Resource quality *B*_1_	Timeliness *B*_11_
			Comprehensiveness *B*_12_
		Course design *B*_2_	Teaching method *B*_21_
			Classroom instruction quality *B*_22_
			Quality of classroom interaction *B*_23_
		Teaching management *B*_3_	Attendance management *B*_31_
			Work published *B*_32_
			Examinations *B*_33_
	Platform management *C*	System Quality *C*_1_	Account security *C*_11_
			Platform stability *C*_12_
		Quality of service *C*_2_	The quality of interaction *C*_21_
			Teaching support *C*_22_

### Determine the Satisfaction Evaluation Level

Online teaching satisfaction evaluation level is defined as *V* = {*v*_1_, *v*_2_, *v*_3_, *v*_4_}={*Verysatisfied*, *satisfied*, *general*, *dissatisfied*}, When *P*(*T*) ∈ [0.90, 1.00], *it is very satisfied v_1_*; when *P*(*T*) ∈ [0.80, 0.90], *it is satisfied v_2_*; when *P*(*T*) ∈ [0.70, 0.80], *it is general v_3_*; when *P*(*T*) ∈ [0.00, 0.70], *it is dissatisfied v_4_*. The online teaching satisfaction questionnaires were analyzed comprehensively. The 9-degree evaluation method evaluates *m* basic-level indexes in combination with the opinions of *n* experts to produce a weighted score. For each basic-level index obtained, the score and the evaluation matrix are shown in Equation 1:


(1) $$D={\left(d_{ij}\right)}_{m\times n}$$

In Equation 1, *d*_*ij*_ is the evaluation of the *i*th index by the *j*th expert.

### Index Probability Calculation

The BN starts from the basic-level index, and proceeds to the middle-level to get the probability of the top-level index.

(1) Prior probability calculation of the basic-level indexes. The calculation formula for the prior probability *d*_*i*_ of the ith basic-level index is shown in Equation 2:


(2) $$d_{i}=\sum_{j=1}^{n}d_{ij}/9n$$

(2) Probability calculation of the middle-level indexes. According to Equation 2, the prior probability vector W of the basic-level index contained in each middle-level index can be calculated, as shown in Equation 3:


(3) $$W={\left(W_{1},W_{2},\ldots ,W_{i},\ldots ,W_{n}\right)}^{T}$$

Where *W*_*i*_ is the prior probability vector element obtained after weighting and normalization of the basic-level indexes by Equations 4, 5:


(4) $$p_{i}=d_{i}\times u_{i}$$

(5) $$W_{i}=p_{i}/\sum_{t=1}^{k}p_{t}$$

Where *u*_*i*_ is the weight of the *i*th basic-level index that constitutes the middle-level index. *k* is the number of basic-level indexes that constitute the middle-level index, and Equation 6 is given:


(6) $$\sum_{i=1}^{k}u_{i}=1$$

A tree Bayesian network, constructed by path selection and prior probability, has a reverse logical causality, and the prior probability of the middle-level index is obtained by the logical operation of its prior probability vector. The logical relation includes two kinds of relation: “AND gate” and “OR gate.” For different relations, the prior probability calculation formulas are also different, as shown in Equations 7, 8:

*A priori* probability calculation of the relationship between “AND gate”:


(7) $$P\left(M=1|N_{1},N_{2},\ldots ,N_{n}=1\right)=\prod_{i=1}^{n}P\left(N_{i}=1\right)$$

*A priori* probability calculation of “OR gate” relation:


(8) $$P\left(M=1|N_{1},N_{2},\ldots ,N_{n}=1\right)=1-\prod_{i=1}^{n}P\left(N_{i}=0\right)$$

In Equations 7, 8, *M* represents the middle-level index, and *Ni* represents the basic-level level index. *P*(*N_*i*_* = 1) represents the prior probability value of the corresponding basic-level level index as *W*_*i*_, while *P*(*Ni* = 0) represents the prior probability value of the corresponding basic-level level index as 1- *W*_*i*_.

(3) Probability calculation of top-level indexes. By calculating the prior probability of the middle-level indexes and judging the corresponding logical relationship of the middle-level indexes, the top-level probability is calculated according to Equation 7, 8. By referring to the defined satisfaction evaluation set *V*, the interval of the top-level probability is found, and the online teaching satisfaction evaluation results can be obtained.

## Case Study

Take a university as an example, the whole university plans to open a total of 2,464 online courses, selected as a typical representative of online teaching.

### Construction of Evaluation Matrix

The online teaching satisfaction questionnaire was issued to the students, and then experts were employed to comprehensively evaluate and score the basic-level indexes, and the basic-level indexes under the same middle-level indexes were weighted to obtain their weighted scores.

According to the scoring results in Table 2, Equation 2 can be used to calculate the prior probability of each basic-level index. Taking learning state *A*_21_ and learning plan *A*_22_ as examples, the prior probability calculation results are *d*_1_ = 0.5242 and *d*_2_ = 0.6889.

**TABLE 2:** Prior probability of evaluation index.

**Indexes**	**Prior probability**	**Indexes**	**Prior probability**
*A*_11_	0.4625	*B*_22_	0.5206
*A*_12_	0.5375	*B*_23_	0.1859
*A*_21_	0.6479	*B*_31_	0.4608
*A*_22_	0.3521	*B*_32_	0.3226
*A*_31_	0.4986	*B*_33_	0.2166
*A*_32_	0.5014	*C*_11_	0.3266
*B*_11_	0.4878	*C*_12_	0.6734
*B*_12_	0.5122	*C*_21_	0.4656
*B*_21_	0.2935	*C*_22_	0.5344

The calculated results of the prior probabilities *P1* and *P2* after weighting in Equation 4 are as follows:


$$p_{1}=d_{1}\times u_{1}=0.5242\times 0.7=0.36694$$

$$p_{2}=d_{2}\times u_{2}=0.6648\times 0.3=0.19944$$

The calculated results of the normalized prior probabilities *W*_*A21*_ and *W*_*A22*_ in Equation 5 are as follows:


$$W_{A_{21}}=\frac{p_{1}}{\sum_{t=1}^{2}p_{t}}=0.36694÷\left(0.366940+19944\right)=0.6479$$

$$W_{A_{22}}=\frac{p_{2}}{\sum_{t=1}^{2}p_{t}}=0.19944÷\left(0.366940+19944\right)=0.3521$$

Similarly, according to the calculated prior probabilities of other basic-level indexes, the weighted and normalized prior probabilities of each basic-level index are shown in Table 2.

Take learning quality *A*_2_ as an example. The calculation result of the prior probability vector *W*_*A_2_*_ is showed as follows:


$$W_{A_{2}}=\left(W_{A_{21}},W_{A_{22}}\right)=\left(0.6479,0.3521\right)$$

Since there is an “AND gate” relationship between *A*_21_ and *A*_22_, the prior probability of *A*_2_ calculated by Equation 7 is 0.2281.


$$P\left(A_{2}\right)=\prod_{i=1}^{2}P\left(N_{i}=1\right)=0.6479\times 0.3521=0.2281$$

Similarly, the prior probabilities of *A_1_,A_3_*, *B*_1_, *B_2_B_3_*, *C*_1_, and *C*_2_ are, respectively, 0.2486, 0.2500, 0.2499, 0.00284, 0.0322, 0.2199, and 0.2488. The prior probabilities of indices *A*, *B*, and *C* were further calculated as 0.5650, 0.2946, and 0.4140.

### Online Teaching Satisfaction Evaluation

The middle-level indexes *A*, *B*, and *C* corresponding to the top-level index form have an “OR gate” relationship. Therefore, Equation 8 is used to calculate the probability of the top-level index, and the calculated result is 0.8205. According to the definition of the satisfaction rating, 0.8202 is in the satisfactory *V*_2_ rating range. Therefore, it is concluded that the degree of online teaching satisfaction at this university is “satisfied”. The result is consistent with the actual satisfaction rating of online teaching. The result shows that the proposed model based on an improved fuzzy BN is effective in online teaching satisfaction evaluation. Therefore, the evaluation results can be used as the basis for the improvement of online teaching.

In view of the online teaching satisfaction evaluation index system, software is used to analyze the influence of the average score of different basic-level indexes on the probability size of top-level indexes. Taking learning environment *A*_11_, learning planning *A*_22_, teaching method *B*_21_, and account security *C*_11_ as examples, the analysis was carried out.

It was found that the influence of the four indices on the top probability increases monotonically with the average score, but their influence on the top probability is different. Among them, the average score of the index learning environment *A*_11_, learning planning *A*_22_, and account security *C*_11_ has a significant impact on the top-level index of online teaching satisfaction *T*, while the low subsection of learning environment *A*_11_ and learning planning *A*_22_ with the average score interval of [0,5] has a severe impact on online teaching satisfaction T. In the average interval of [5,9], the influence is relatively gentle. This indicates that in the low subsections of [0,5] because of the students’ poor learning environment and unscientific learning plan, there are serious restrictions on the improvement of online teaching satisfaction. Therefore, in this case, other aspects can be considered for improvement, so as to further improve online teaching satisfaction. The influence of account security *C*_11_ on online teaching satisfaction *T* has been increasing. It can be seen that account security has an important influence on the rapid improvement of online teaching satisfaction. The more secure the account, the higher the satisfaction of online teaching. However, teaching method *B*_21_ has a gentle influence on the top-level index *T* of online teaching satisfaction. Students can make efficient self-adjustment to adapt to their teachers’ teaching methods and have a high acceptance of their teachers’ teaching methods. Therefore, in order to improve the satisfaction of online teaching, we can focus on improving students’ learning environment, learning state, and the security of their accounts.

### Analysis of Online Teaching System

After calculation, the probability value of the top-level index *T* of online teaching satisfaction is 0.8205, which belongs to the degree of “*satisfied.*” However, there are still defects and deficiencies in online teaching. Based on the above research results, in order to improve online teaching satisfaction, the following suggestions are put forward.

(1) Improving students’ learning environment. A good learning environment is the basis for teachers and students to conduct online teaching. Schools and platforms should invest more in hardware construction. More backup equipment and solutions should be available in case of network congestion. It is suggested that universities should arrange and adjust the online teaching time and implement online teaching at different peaks. According to the needs of different courses and features of the platform, different online teaching strategies are selected. According to the survey data, the performance of Tencent meeting, pinning, and learning pass are stable. Students and teachers have high satisfaction when using them. At the same time, the teachers should prepare for the online teaching environment in advance, such as live broadcasting and recording. In order to ensure normal condition online teaching, the teachers should adjust the teaching content according to the software and hardware conditions. Online teaching satisfaction is naturally improved when the learning environment is reliable.

(2) A clear study plan. Students’ independent learning ability is required to be higher with online teaching. Students should make a reasonable study plan firstly. Then they must study hard and review in time. They should arrange learning time reasonably and strive to improve their learning efficiency. According to the teaching objectives, teachers should scientifically formulate the course learning outline. They should provide the relevant preview materials, thinking questions, and related online course links to the students in advance, so as to guide the students to realize their self-oriented deep learning. Clarifying learning planning is a key step to achieving satisfactory learning outcomes. It is an important index to measure the satisfaction of online teaching. Therefore, teachers should help their students to make a scientific learning plan, which can effectively improve the satisfaction of online teaching.

(3) Improve classroom interaction. Effective online teaching should be a learning process that involves the participation of all students and the interactions between teachers and students. So the interactions among teachers and students, and between students and students can form multiple interactive modes of online teaching. Teachers can discuss with students with the help of cloud class, nail, learning pass, etc., after online class. They can also set time-limited online tests to increase real-time comment results. If some course assistants can be hired, they collect and feedback students’ problems in the process of online teaching. And they will cooperate with the teachers to better complete online teaching. In addition, students’ independent ability would be constantly cultivated through lively classroom interaction. Participatory teaching is good for improving students’ initiative and interest in learning. Teachers and students will have a better experience of online teaching with good classroom interactions. From the perspective of emotion analysis, the teachers communicate the useful course information to students in time by bullet screen. This method will build a bridge of interaction between teachers and students. The emotional connection between teachers and students will be further deepened, so that the satisfaction of online teaching is improved.

(4) Develop a new assessment mechanism. Online teaching assessment mechanisms are an important link in the evaluation of online teaching management. The scientific and reasonable construction of online teaching assessment systems is conducive to improving teachers’ initiative in teaching. The final examination plays a decisive role in the assessment of students’ grades. They study hard only for a few weeks before the final exam. This kind of cramming learning method does not help to master their knowledge. As a result, most students are slack in their studies. Based on the above problems, a new assessment mechanism is proposed by changing the composition of grades and defining their total score as “the average score accounts for 60%, the final score accounts for 40%” (or “the average score accounts for 70%, the final score accounts for 30%”). The average score has a large impact on the total score, which means the assessment mechanism can run through the whole process of online teaching. This kind of comprehensive evaluation focuses on the evaluation of students’ learning process including the students’ participation in class, the status of homework completion, the number of discussion posts, and the final exam results. This new assessment mechanism can avoid students from slacking off in class for most of the semester, and only make a brief effort at the end of the semester. It requires students to take online classes seriously, actively perform, and earnestly complete learning tasks. This new assessment mechanism can better play the role of the teachers, so as to improve the satisfaction of online teaching.

## Data Availability Statement

The original contributions presented in the study are included in the article/supplementary material, further inquiries can be directed to the corresponding author.

## Ethics Statement

The studies involving human participants were reviewed and approved by the Ethics Committee of Wuxi Institute of Technology. Written informed consent to participate in this study was provided by the participants.

## Author Contributions

The author confirms being the sole contributor of this work and has approved it for publication.

## Conflict of Interest

The author declares that the research was conducted in the absence of any commercial or financial relationships that could be construed as a potential conflict of interest.

## Publisher’s Note

All claims expressed in this article are solely those of the authors and do not necessarily represent those of their affiliated organizations, or those of the publisher, the editors and the reviewers. Any product that may be evaluated in this article, or claim that may be made by its manufacturer, is not guaranteed or endorsed by the publisher.
